# Pyoderma gangrenosum treated with oral abrocitinib in a 54-year-old woman: A case report

**DOI:** 10.1016/j.jdcr.2025.02.022

**Published:** 2025-03-10

**Authors:** Martin Moises E. Estrella, Vermén M. Verallo-Rowell

**Affiliations:** a Resident, Department of Dermatology, Makati Medical Center, Makati, Philippines; b Active Consultant, Department of Dermatology, Makati Medical Center, Makati, Philippines

**Keywords:** abrocitinib, JAK kinase inhibitor, Philippines, pyoderma gangrenosum, treatment

## Introduction

Pyoderma gangrenosum is a debilitating skin disease marked by idiopathic neutrophil infiltration that causes the destruction of tissue and ulceration. Currently, there are no international treatment guidelines. Abrocitinib, a Janus Kinase (JAK)1 selective inhibitor, has been used as a novel treatment in a few case reports. We present a case of a 54-year-old female who presented with a solitary, markedly inflamed, rapidly widening ulcer on the right lateral ankle successfully treated with light debridement followed by barrier-repair topicals and abrocitinib.

## Case report

This 54-year-old British-Filipino female, diagnosed for 20 years with ulcerative colitis and mostly treated with Chinese herbals, occasionally experienced episodes of minimal abdominal cramps and loose stools. She was referred to dermatology service for a solitary painful ulcer on the right lateral ankle with swelling of the right lower leg.

Four months before referral, the patient experienced muscle tightness in both lower extremities with edema and extreme pain in the right ankle, the right lateral side of which developed erythematous and edematous plaques with a solitary fluid-filled bulla. She was prescribed cephalexin 500 mg/capsule, 1 capsule every 6 hours for 7 days, and advised ice compress application and frequent deep massaging of the right lower leg, which she followed.

The right ankle bulla ruptured into a large ulcer with a purulent and hemorrhagic discharge, for which an emergency consult was done. A diagnosis of cellulitis led to an in-patient admission. Clindamycin 600 mg every 6 hours and intravenous levofloxacin 750 mg every 24 hours were given. Wound debridement, exploration, and application of vacuum-assisted closure dressing of the right foot were done by the orthopedic surgeon on the third hospital day. Culture and sensitivity of the wound taken prior to the antibiotic revealed no microorganisms.

**Fig 1:**
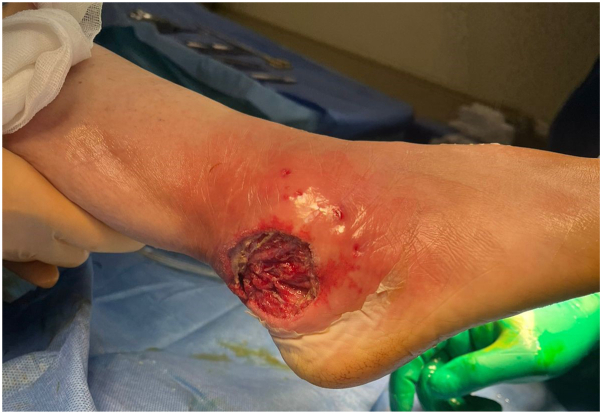
Day 3 upon referral. Right lateral ankle 10 × 8 cm diffusely erythematous swelling and central ulcer.

A referral to dermatology service was done on the fifth hospital day. The 10 × 8 cm right lateral ankle ulcer was brightly erythematous, with reddish to violaceous borders, a ragged, undermined edge, and a wide area of erythema around the ulcer (Fig 1).

The diagnosis of a bullous variant of pyoderma gangrenosum was made based on the 20-year history, the rapid development of a bulla evolving into the ulcer, and a negative bacterial culture. A biopsy was not performed as the patient refused due to the pain and extent of the open wound. When a biopsy is not done, the PARACELSUS score offers a comprehensive and practical diagnostic system. A total score of 10 points or higher signifies a high likelihood of pyoderma gangrenosum, which helps distinguish it from venous leg ulcers.^1^ This patient scored a total of 16 points (Table I).

**Table I:** PARACELSUS scoring criteria,^1^ patient score, and overall assessment

Diagnostic criteria	Point values	Patient score
Progressive course of disease	3	3
Assessment (absence) of relevant differential diagnoses	3	0
Reddish-violaceous wound margin	3	3
Amelioration (alleviation) by immunosuppressant drugs	2	2
Characteristically bizarre ulcer shape	2	2
Extreme pain (>4 visual analog scale)	2	2
Localized pathergy phenomenon (localization of lesion at the site of trauma)	2	2
Suppurative inflammation in histopathology	1	0
Undermined wound margin	1	1
Systemic disease associated	1	1
Total	20	16
Assessment	≥10 = highly likely <10 = unlikely	Pyoderma gangrenosum highly likely

Patient score of 16 indicates that pyoderma gangrenosum is highly likely.

**Fig 2:**
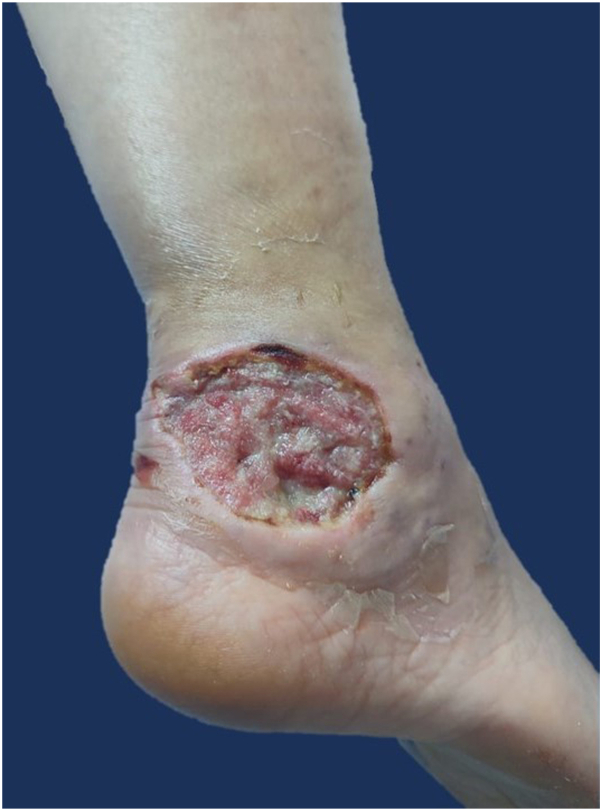
Clinical picture upon discharge. Right lateral ankle 3.7 × 5.7 cm solitary pinkish ulcer, with contracting borders.

Wound care was done by light cleansing of the wound with cold-pressed virgin coconut oil (CP-VCO)-soaked gauze, gentle removal of wound and edge scabs, application of 30% trichloroacetic acid on the rounded edges of the ulcer, and then covered with a CP-VCO-saturated gauze dressing. This oil was chosen for its effects on faster re-epithelialization, protective barrier functions, as well as antimicrobial, antiviral, and antifungal activity, as discussed in a review by Lin et al (2018).^2^ The dressing was occluded with a thin plastic and an elastic wrap. Abrocitinib 200 mg per tablet was started once a day on day 4. The patient noted less pain and redness after 2-3 days, with decreasing ulcer size. Seven days after admission, she was discharged with a pinkish, no longer bright red ulcer, with contracting borders, measuring 3.7 × 5.7 cm (Fig 2).

The first month of outpatient wound care consisted of twice a week CP-VCO cleansing, light removal of scabs, 35% trichloroacetic acid applied on the ulcer edges, followed by the CP-VCO-soaked gauze and elastic rolled bandage dressing. Abrocitinib at 200 mg a day was maintained.

**Fig 3:**
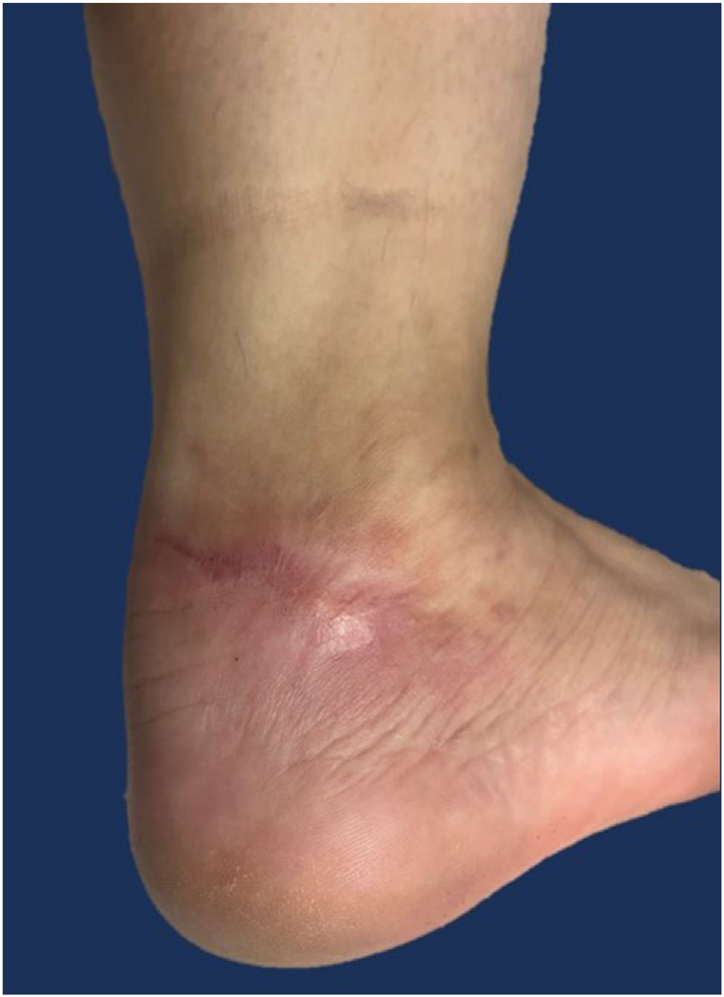
Clinical picture after 4 months of treatment. Solitary 1.8 × 4 cm pinkish to skin-colored linear thin scar on the right lateral ankle.

During the second to fourth month of treatment, wound care was done using the same regimen, with an added pharmacist-compounding of 4% monolaurin, 1% monocaprin, and 10% tranexamic acid in a petrolatum base, applied twice a day on the immediate surrounding reddish areas. This was used for reducing postinflammatory hyperpigmentation and improving skin hydration.^2^^,^^3^ At the end of the fourth month, the ulcer completely healed into a solitary pinkish to skin-colored linear scar measuring 1.8 × 4 cm (Fig 3). While on abrocitinib, abdominal cramps and loose stools decreased significantly. There were no side effects noted. As the patient was scheduled to leave overseas a day after the last follow-up, she was advised to maintain the current dose of abrocitinib as well as to see her physician abroad and then show our treatment summary as the basis for the maintenance dosage.

## Discussion

The suggested pathophysiology of pyoderma gangrenosum involves the interaction of innate and adaptive immunities and a state of neutrophil-driven autoinflammation. Since there are no international pyoderma gangrenosum guidelines of care, therapeutic options consist of wound care, topical and oral corticosteroids, calcineurin inhibitors, and intralesional corticosteroids.^4^ Tan and Tolkachjov (2024) reported that pyoderma gangrenosum lesions exhibit elevated levels of proinflammatory molecules, including various cytokines, interleukins, JAK-1, JAK-2, JAK-3, and interferon gamma. JAK inhibitors, classified under level 4 evidence, target these pathways and have shown promise in treatment. As more case reports document successful outcomes, the use of JAK inhibitors for this condition is expected to increase, potentially strengthening the level of evidence over time.^5^

Chen et al (2024) published a case report of a patient with painful ulcerative lesions on the perianal area, diagnosed with pyoderma gangrenosum, and initially unresponsive to doxycycline, isotretinoin, glucocorticoids, and cyclosporine. Oral abrocitinib given at 100 mg daily resulted in improvement of the redness and symptoms after a week with near-complete resolution of the pain and swelling and reduced depth and area of the ulcer after 4 weeks.^6^ This study, along with the drug’s ready availability, led to the selection of abroticinib over prednisone as the patient preferred a fast response and improvement before returning home.

This case report is of a patient with a typical presentation of bullous pyoderma gangrenosum, successfully treated with a combination of gentle wound care, barrier repair topical medications, and oral abrocitinib. The excellent results of this case report highlight the opportunity to consider a combination of treatment modalities for future clinical use.

## Conflicts of interest

None disclosed.
